# 130. Influencing Antibiotic Prescribing for Patients with *Enterobacter cloacae* or *Klebsiella aerogenes* by Use of Templated Comments

**DOI:** 10.1093/ofid/ofad500.203

**Published:** 2023-11-27

**Authors:** Anna Landolt, Nicholas Bennett, Laura Aragon, Sarah E Boyd, Cindy Essmyer, Matthew Humphrey

**Affiliations:** Saint Luke's Health System, Kansas City, Missouri; Saint Luke's Health System, Kansas City, Missouri; Saint Luke's Health System, Kansas City, Missouri; Saint Luke's Health System, Kansas City, Missouri; Saint Luke's Health System, Kansas City, Missouri; University of Kansas Health System, Kansas City, Missouri

## Abstract

**Background:**

Antimicrobial resistance poses a significant threat in treating bacterial infections. One such concern is the treatment of AmpC beta-lactamase producing *Enterobacterales*. In October 2019, Saint Luke’s Health System incorporated an automated microbiology comment advising the avoidance of third generation cephalosporins in patients with cultures positive for two bacteria which most frequently express AmpC resistance: *Enterobacter cloacae* and *Klebsiella aerogenes*. We assessed the comments effect on treatment decisions and microbiologic outcomes.

**Methods:**

This is a retrospective, pre-post, cohort study in a single health system. Patients were included if they were inpatients 18 years of age or older and had urine or blood cultures positive for *E. cloacae* or *K. aerogenes*. Patients were excluded if cultures were determined to be colonizing pathogens. Pre- (May to December 2018) and post-comment (May to December 2020) cohorts were compared to assess the influence of the comment on therapy selection and subsequent development of resistance. The primary endpoint was escalation to targeted antimicrobial therapy within the first 24 hours after culture speciation. Secondary outcomes included escalation within 72 hours of culture speciation, time to escalation, length of hospital stay, and subsequent development of multi-drug resistant organism one year following admission.

**Results:**

Escalation of targeted antimicrobial therapy within 24 hours of culture speciation occurred in 20 patients in the pre-implementation group and 46 patients in the post-implementation group (p< 0.001). Escalation within 72 hours occurred in 34 patients in the pre-implementation group and 57 patients in the post-implementation group (p=0.004). Median time to change in antimicrobial therapy was 23 hours in the pre-implementation group and 7 hours in the post-implementation group (p< 0.001). There was no difference between groups in development of MDR (5 vs. 5.7%, p=0.6).

**Conclusion:**

Implementation of templated microbiology comments increased the rate of escalation to targeted antimicrobial therapy in hospitalized patients with blood or urine cultures positive for *E. cloacae* or *K. aerogenes.* Templated comments are an effective, minimally invasive strategy to promote antimicrobial stewardship.

**Disclosures:**

**All Authors**: No reported disclosures

